# Genome-wide allele frequency studies in Pacific oyster families identify candidate genes for tolerance to ostreid herpesvirus 1 (OsHV-1)

**DOI:** 10.1186/s12864-023-09744-0

**Published:** 2023-10-23

**Authors:** Konstantin Divilov, Noah Merz, Blaine Schoolfield, Timothy J. Green, Chris Langdon

**Affiliations:** 1 Department of Fisheries, Wildlife, and Conservation Sciences, Coastal Oregon Marine Experiment Station, Oregon State University, Hatfield Marine Science Center, Newport, OR 97365 USA; 2 Centre for Shellfish Research, Vancouver Island University, Nanaimo, BC V9R 5S5 Canada

**Keywords:** QTL, GWAFS, Disease, Mortality, Virus

## Abstract

**Background:**

Host genetics influences the development of infectious diseases in many agricultural animal species. Identifying genes associated with disease development has the potential to make selective breeding for disease tolerance more likely to succeed through the selection of different genes in diverse signaling pathways. In this study, four families of Pacific oysters (*Crassostrea gigas*) were identified to be segregating for a quantitative trait locus (QTL) on chromosome 8. This QTL was previously found to be associated with basal antiviral gene expression and survival to ostreid herpesvirus 1 (OsHV-1) mortality events in Tomales Bay, California. Individuals from these four families were phenotyped and genotyped in an attempt to find candidate genes associated with the QTL on chromosome 8.

**Results:**

Genome-wide allele frequencies of oysters from each family prior to being planting in Tomales Bay were compared with the allele frequencies of oysters from respective families that survived an OsHV-1 mortality event. Six significant unique QTL were identified in two families in these genome-wide allele frequency studies, all of which were located on chromosome 8. Three QTL were assigned to candidate genes (*ABCA1*, *PIK3R1*, and *WBP2*) that have been previously associated with antiviral innate immunity in vertebrates.

**Conclusion:**

The identification of vertebrate antiviral innate immunity genes as candidate genes involved in molluscan antiviral innate immunity reinforces the similarities between the innate immune systems of these two groups. Causal variant identification in these candidate genes will enable future functional studies of these genes in an effort to better understand their antiviral modes of action.

**Supplementary Information:**

The online version contains supplementary material available at 10.1186/s12864-023-09744-0.

## Background

Ostreid herpesvirus 1 (OsHV-1) is a viral pathogen that causes disease and mortality in Pacific oysters (*Crassostrea gigas*) [1]. Since 2008, the emergence of virulent variants of OsHV-1 in Australia [2], France [3], New Zealand [4], and the United States [5] have led to the development of strategies to mitigate the negative economic effects from massive mortalities on oyster growers. One strategy that has been uniformly adopted by farmers in these countries is the use of oysters that are tolerant to OsHV-1 [6–9]. However, despite the success of breeding oysters with OsHV-1 tolerance, the genes and underlying mechanisms responsible for this increased tolerance have yet to be determined.

OsHV-1 is in the *Herpesvirales* order, which contains herpesviruses that infect and cause disease in a wide range of animal hosts spanning mammalian vertebrates, non-mammalian vertebrates, and invertebrates [10]. Genome-wide association studies (GWAS) in humans [11, 12], mice [13], rats [14], horses [15], pigs [16], chickens [17], common carp [18, 19], and Pacific oysters [20–22] have found significant regions in their respective genomes that are associated with herpesvirus disease severity. Many GWAS studies have been conducted in animals of agricultural importance because genetic loci associated with herpesvirus tolerance can be used for selective breeding to reduce herpesvirus-associated disease.

Selective breeding for a genetic locus controlling survival to OsHV-1, as well as antiviral gene expression, has recently been conducted in a Pacific oyster population in the United States [20]; however, the significance interval of this locus on chromosome 8 of the Pacific oyster genome spanned 6.8 Mb and contained 316 protein-coding genes. Identifying the genes responsible for the increased tolerance to OsHV-1 in this locus could provide insights into the mechanisms behind this trait. The objectives of the current study were to find candidate genes on chromosome 8 responsible for OsHV-1 tolerance in four biparental families (Table 1) from the Molluscan Broodstock Program (MBP) breeding population [23] by comparing the genome-wide allele frequencies of oysters that survived an OsHV-1 mortality event in Tomales Bay, California, with oysters that were collected prior to planting.

**Table 1:** Family sample and SNP statistics. Oysters from four biparental families were randomly sampled prior to being planted in Tomales Bay, California, and all planted oysters that survived an OsHV-1-associated mortality event were sampled. Sampled oysters were genotyped to obtain SNPs on the maternal and paternal haplotypes that were used in genome-wide allele frequency studies (GWAFS)

Family	Oysters (pre-planting samples)	Oysters (planted in Tomales Bay)	Oysters (post-mortality samples)	SNPs
30.004	166	Replicate 1: 150 Replicate 2: 150 Replicate 3: 150	Replicate 1: 95 Replicate 2: 133 Replicate 3: 124	Maternal haplotype: 16,794 Paternal haplotype: 17,165
30.058	174	Replicate 1: 150 Replicate 2: 150 Replicate 3: 150	Replicate 1: 49 Replicate 2: 42 Replicate 3: 54	Maternal haplotype: 18,381 Paternal haplotype: 18,097
30.062	145	Replicate 1: 150 Replicate 2: 150 Replicate 3: 150	Replicate 1: 105 Replicate 2: 82 Replicate 3: 68	Maternal haplotype: 18,095 Paternal haplotype: 18,221
30.065	89	Replicate 1: 150 Replicate 2: 150 Replicate 3: 150	Replicate 1: 106 Replicate 2: 96 Replicate 3: 79	Maternal haplotype: 18,060 Paternal haplotype: 17,956

## Results

**Fig. 1:**
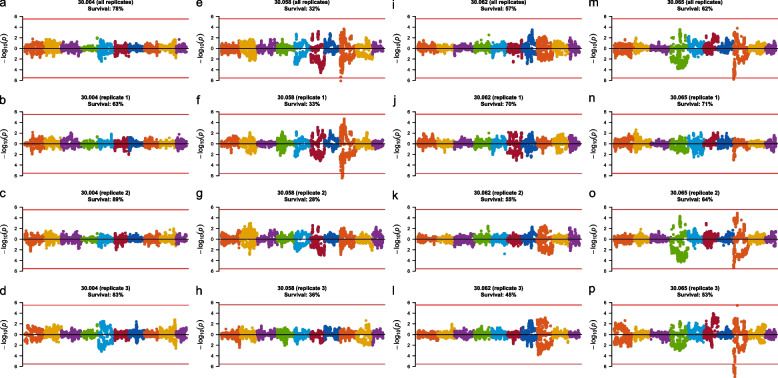
Manhattan plots showing the results of the genome-wide allele frequency studies (GWAFS) for survival to an OsHV-1-associated mortality event in Tomales Bay in four biparental families. GWAFS were performed with the post-mortality group as either oysters from all three replicate cages or oysters from one of the three replicate cages. Chromosomes 1 to 10 are indicated by different colors from left to right. Log-transformed *p*-values above and below zero represent those associated with the maternal and paternal haplotypes, respectively. The red horizontal line represents the genome-wide Bonferroni-corrected *p*-value significance threshold of 0.05

**Fig. 2:**
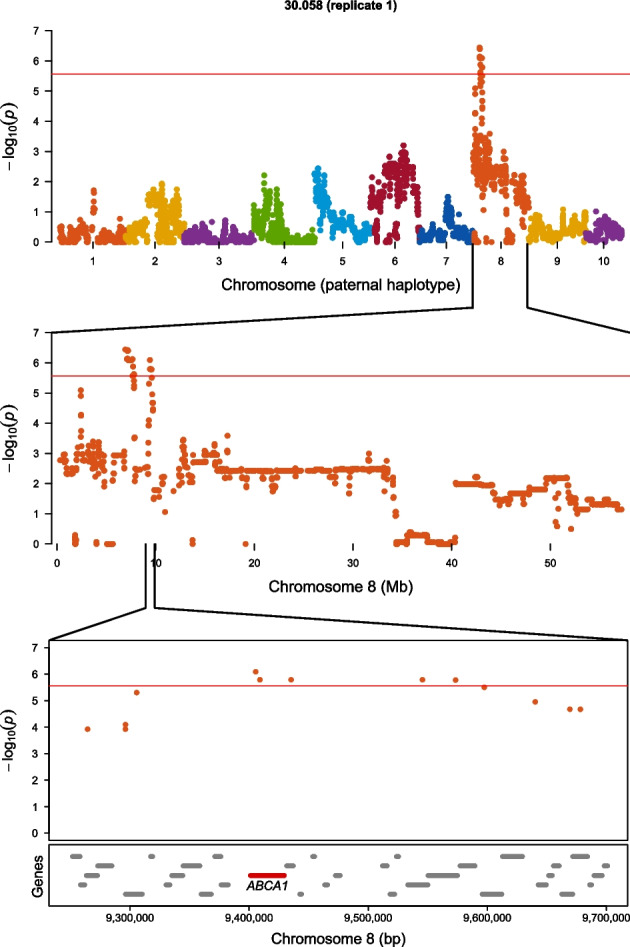
Genome-wide allele frequency study of the paternal haplotype of family 30.058 (replicate 1) at the **a** genome, **b** chromosome, and **c** sub-chromosome levels. The red horizontal line represents the genome-wide Bonferroni-corrected *p*-value significance threshold of 0.05. The gene highlighted in red is the candidate gene assigned to the QTL while the gray colored genes are other genes in the region

**Fig. 3:**
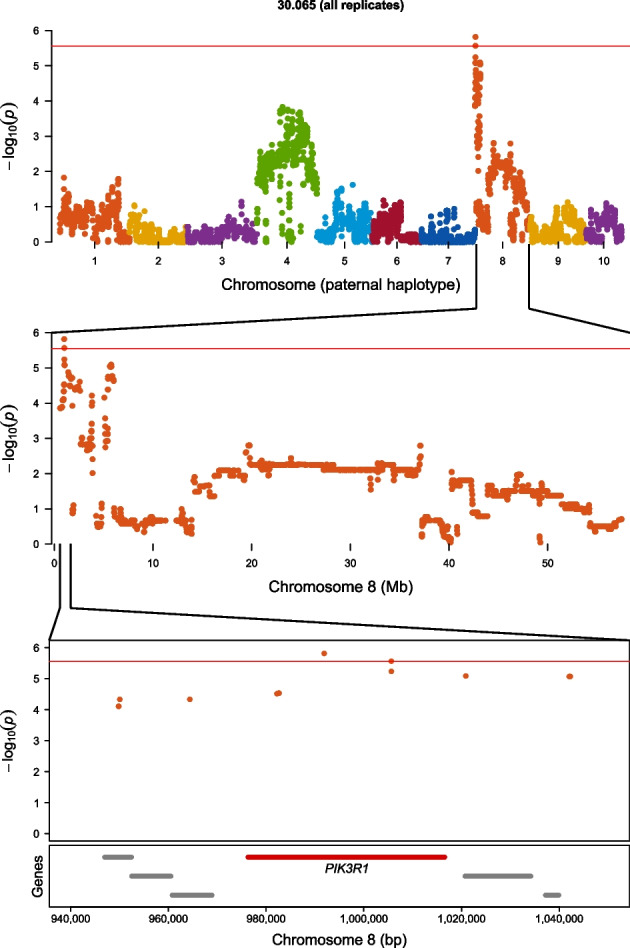
Genome-wide allele frequency study of the paternal haplotype of family 30.065 (all replicates) at the **a** genome, **b** chromosome, and **c** sub-chromosome levels. The red horizontal line represents the genome-wide Bonferroni-corrected *p*-value significance threshold of 0.05. The gene highlighted in red is the candidate gene assigned to the QTL while the gray colored genes are other genes in the region

Six significant QTL were identified from the genome-wide allele frequency studies (GWAFS) in two families that mapped to unique positions on chromosome 8 in the genome (Table 2; Fig. 1). The GWAFS of family 30.058 identified three QTL on chromosome 8. The GWAFS of family 30.065 identified four unique QTL on chromosome 8, one of which mapped to the same genomic region as a QTL found in the GWAFS with family 30.058. The GWAFS of families 30.004 and 30.062 identified no QTL. Four candidate genes were assigned to QTL on chromosome 8 and included homologs to *ABCA1* (phospholipid-transporting ATPase ABCA1) (Fig. 2), *FRRS1* (ferric-chelate reductase 1), *PIK3R1* (phosphatidylinositol 3-kinase regulatory subunit alpha) (Fig. 3), and *WBP2* (WW domain-binding protein 2).

**Table 2:** QTL for survival to an OsHV-1-associated mortality event in Tomales Bay identified in two biparental families from cohort 30 in genome-wide allele frequency studies (GWAFS) with the post-mortality group as either oysters from all three replicate cages or oysters from one of the three replicate cages

Family	Haplotype	Chr	Position (bp)^a^	Δp^b^	Candidate gene
30.065 (all replicates)	Paternal	8	991,891–991,891–1,005,626	0.31	*PIK3R1*
30.065 (replicate 3)	Paternal	8	991,891–1,404,593–1,810,572	0.35	—
30.065 (replicate 3)	Paternal	8	2,087,539–2,604,508	0.34	—
30.058 (all replicates)	Paternal	8	2,403,628	0.28	—
30.065 (replicate 3)	Paternal	8	5,061,600	0.43	*WBP2*
30.065 (replicate 3)	Paternal	8	5,413,906–5,634,130–6,008,157	0.25	—
30.058 (replicate 1)	Paternal	8	6,911,780–6,911,780–7,788,427	0.37	*FRRS1*
30.058 (replicate 1)	Paternal	8	9,405,510–9,405,510–9,573,362	0.35	*ABCA1*

^a^Positions represent the first significant, most significant, and last significant SNPs in a QTL interval. When only two positions are provided, they are the first and last SNPs of an interval containing two or more SNPs that have tied for being the most significant SNP. When only one position is provided, it represents a QTL with only one significant SNP

^b^Change in allele frequency between the pre-planting and post-mortality groups of oysters

GWAFS using post-mortality oysters from all replicates did not always find the same QTL as GWAFS using post-mortality oysters from individual replicates; for example, the GWAFS of family 30.058 using oysters from all replicates did not find the same QTL as the GWAFS of family 30.058 using oysters from only replicate 1 (Table 2). Additionally, different QTL were present or absent when using oysters from different replicate cages within the same family in a GWAFS; for example, significant QTL on chromosome 8 in family 30.065 were found in the GWAFS using oysters from replicate 3 (Fig. 1p) but not when oysters from replicates 1 and 2 were used (Fig. 1n, o). The presence of QTL in a GWAFS using oysters from a replicate was not always determined by the percentage of oysters surviving in that replicate; for example, 30.062 (replicate 2) and 30.065 (replicate 3) had 55% and 53% survival in the field, respectively, but the GWAFS with the former found no QTL (Fig. 1k) while the GWAFS with the latter found four QTL (Fig. 1p).

## Discussion

The QTL found in the GWAFS were all located on one end of chromosome 8 where an antiviral QTL was previously identified in a GWAS with oyster families from MBP cohorts 27 and 29 [20]. With the greater mapping resolution available in the current study, it is evident that there are multiple QTL segregating on chromosome 8 rather than a single QTL segregating in the MBP population. In the field study, there was an unexpected amount of variability in the presence and absence of QTL in GWAFS with oysters from different replicate cages. Because there was a limit to how many oysters were able to be put into a single replicate cage without density-dependent mortality, oysters from a single family were distributed among multiple replicate cages in order to increase the sample size in the GWAFS. We found that GWAFS with oysters from different replicates from the same family that experienced similar levels of mortality did not necessarily identify the same QTL despite the replicates being planted very close to each other; for example, the three replicate cages of family 30.058 were planted approximately 1 m from each other and experienced between 64 and 72% mortality but a QTL was only detected in the GWAFS with replicate 1. Factors other than host genetics, such as size [24, 25], energy reserves [26], and opportunistic bacteria [27], have been shown to play a role in survival to OsHV-1. Additionally, significant micro-scale spatial effects on survival have previously been observed during an OsHV-1 mortality event that have been associated with OsHV-1 load heterogeneity [28, 29]. We believe that these factors were likely responsible for the presence/absence variability of QTL in the GWAFS. Ideally, oysters within cages would have been monitored daily to obtain a continuous survival phenotype, e.g., time to death, as well as other phenotypes, such as size, to better understand the influence of other potentially interacting factors; however, the planting site in this study was intertidal and accessible only at low tide with a boat, which made trips to the site for long-term, short-interval sampling impractical for the company hosting the study site.

Three candidate genes (*ABCA1*, *PIK3R1*, *WBP2*) identified among the QTL have previously been associated with the pathogenesis of herpesviruses or other viruses. *ABCA1* is a regulator of cholesterol and has been associated with the severity of herpes simplex virus 2 in a GWAS in humans [30]. Additionally, *ABCA1* gene expression has been shown to be upregulated after infection with Marek's disease virus (gallid alphaherpesvirus 2) in chicken fibroblasts [31]. *PIK3R1* is part of the PI3K/Akt/mTOR signaling pathway, which is involved in innate immunity [32], and mutations in *PIK3R1* have been associated with severe, recurrent, or persistent infections of herpesviruses in humans [33]. *WBP2* is a gene involved in many signaling pathways, including the PI3K/Akt/mTOR signaling pathway [34], and has been shown to be downregulated in pig macrophages after infection with porcine reproductive and respiratory syndrome virus [35].

The identification of candidate genes in the Pacific oyster associated with survival to an OsHV-1 mortality event that have previously been implicated in the pathogenesis of vertebrate herpesviruses reinforces the parallels previously identified between the innate immune systems of molluscs and vertebrates [36, 37]. It also suggests that OsHV-1 could potentially be used as a model to study vertebrate herpesviruses, some of which share the genomic structure of OsHV-1 [10], and that insights from vertebrate herpesviruses could potentially be used to better understand OsHV-1. Despite identifying candidate genes for some of the QTL, it was not possible to identify candidate causal variants related to these candidate genes with the reduced-representation sequencing method used in this study, i.e., GBS. We anticipate that future studies utilizing whole genome sequencing will enable the identification of candidate causal variants for these genes and that these variants will motivate future functional studies of these genes.

## Methods

### Genotyping

Families in cohort 30 of the Molluscan Broodstock Program (MBP) were reared, planted in Tomales Bay, California, and phenotyped for survival as previously described by Divilov et al. [20]. Briefly, cohort 30 (*n* = 79 biparental families) was spawned in the MBP hatchery at the Hatfield Marine Science Center (HMSC) in Newport, Oregon, on 18 March 2021 and planted in Tomales Bay, California (38°12′17″N 122°56′05″W) on 8 June 2021. Oysters were checked every two weeks until the detection of significant mortality. Oyster mortality counts were taken on 19 October 2021. Individuals in four of these families, namely families 30.004, 30.058, 30.062, 30.065 (pedigree provided in Fig. S1), were chosen for pre-planting and post-mortality genotyping. Individuals in these families were chosen because their parents were heterozygous for a SNP at position 9,719,736 on chromosome 8, which has been associated with survival in Tomales Bay and basal antiviral gene expression [20]. Whole animal tissues of spat (juvenile oysters) from the four families obtained from the same pool of spat that were chosen for planting in Tomales Bay were stored in 95% ethanol and are referred to as the pre-planting samples (Table 1). These four families were planted in Tomales Bay in triplicate with a density of 150 oysters per replicate cage that was concurrent with the planting date and location of cohort 30 in Tomales Bay described above. The replicate cages for this additional planting were planted adjacent to each other in a row. Mantle tissue from oysters that survived the mortality event in each replicate cage from the four families were sampled in a biosafety level 2 laboratory, stored in 95% ethanol, and are referred to as the post-mortality samples (Table 1).

DNA from the pre-planting and post-mortality samples as well as the parents of the four families were extracted using the MagMAX DNA Multi-Sample Ultra 2.0 Kit (Thermo Fisher Scientific) and quantified using the Quant-iT Broad Range dsDNA Assay Kit (Thermo Fisher Scientific) on a Synergy LX Multi-Mode Microplate Reader (BioTek). Barcoded 96-plex genotyping-by-sequencing (GBS) libraries were constructed as described by Elshire et al. [38] using the ApeKI restriction enzyme and sequenced on an Illumina NextSeq 2000 (1 × 100 bp) at Oregon State University’s Center for Quantitative Life Sciences.

Reads were demultiplexed using Axe [39] and adapter trimmed using fastp [40]. A 5’ to 3’ sliding window of 4 bp was also used in fastp to drop all base pairs after the mean quality dropped below 20. Afterward, all reads less than 50 bp were removed. Filtered reads were then aligned to the *Crassostrea gigas* NCBI RefSeq genome [41] using bwa [42], and biallelic SNPs were called using bcftools [43] with a minimum mapping quality of 40 and a minimum base quality of 30. SNPs were then phased and imputed using AlphaFamImpute [44] with the genotype calling threshold set to 0.9 after filtering for minimum read depth (< 5), maximum read depth (> 100), minor allele frequency (< 0.15), and SNP missingness rate (> 0.8) (Table 1). Parentage assignment of offspring in each of the four families was confirmed using the *apparent* algorithm [45].

### Genome-wide allele frequency study (GWAFS)

Fisher’s exact test [46] was used to test for SNP allele frequency differences between the pre-planting and post-mortality samples within a family. Tests were conducted using SNPs within maternal and paternal haplotypes in a family. Additionally, tests were performed with the post-mortality samples being either oysters from all three replicate cages of a family or oysters from one of the three replicate cages of a family. A Bonferroni-corrected *p*-value of 0.05 was used as the genome-wide significance threshold. The R packages *fastman* [47] and *LocusZoom-like* [48] were used to generate Manhattan plots, and the RefSeq genome annotation was used to identify candidate genes.

### Supplementary Information

**Additional file 1:** **Fig. S1.** Pedigree of families 30.004, 30.058, 30.062, and 30.065. Ancestry prior to cohort 22 is not shown as families in these cohorts were not spawned using single pair matings.

## Data Availability

The raw GBS reads of the oysters used in this study have been deposited at NCBI (BioProject PRJNA873124). The code used to run the analyses is available at https://github.com/kdivilov/BMC_Genomics_2023.
