# Supplementary material for: Genome-wide allele frequency studies in Pacific oyster families identify candidate genes for tolerance to ostreid herpesvirus 1 (OsHV-1)
Source: BMC Genomics. 2023 Oct 23;24:631. doi: 10.1186/s12864-023-09744-0 (PMC10594793; doi:10.1186/s12864-023-09744-0)
Supplement: Supplementary file 1 — Additional file 1: Fig. S1. Pedigree of families 30.004, 30.058, 30.062, and 30.065. Ancestry prior to cohort 22 is not shown as families in these cohorts were not spawned using single pair matings. [file 12864_2023_9744_MOESM1_ESM.docx]

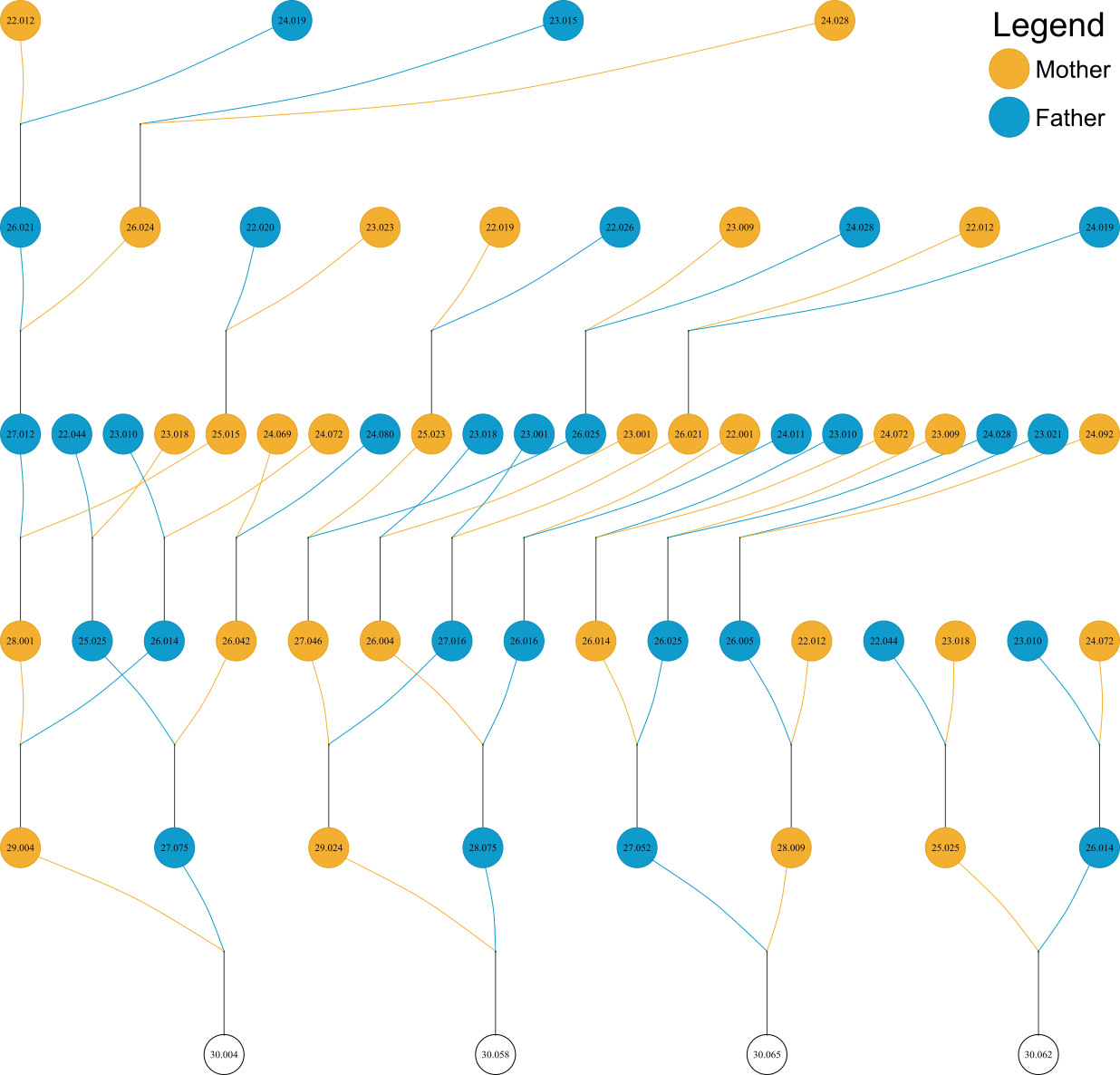


Fig. S1. Pedigree of families 30.004, 30.058, 30.062, and 30.065. Ancestry prior to cohort 22 is not shown as families in these cohorts were not spawned using single pair matings.
